# Effects of rational-emotive health education program on hiv risk perceptions among in-school adolescents in nigeria: Erratum

**DOI:** 10.1097/MD.0000000000009317

**Published:** 2017-12-15

In the article, “Effects of Rational-Emotive Health Education Program on HIV risk perceptions among in-school adolescents in Nigeria”,^[1]^ published Volume 95 Issue 29 of *Medicine*, the authors have erroneously described the instrument for data collection as an 8-items questionnaire instead of the 18-items that were used. This correction applies to the whole article.

In Section 2.3 Procedure, the last statement “the current study's intervention program sessions lasted for 40 minutes or more and twice per week” should appear as 45 minutes.

In Section 2.4.1, the statement “higher scores on the scale are positively associated with high risk. As part of the adaptation......” should be removed.

In addition, the original Table at the Appendix should be removed. The authors had described the measure as a 5-point scale in the method section of the manuscript but what was in the Appendix was a 4-point scale. A response option which ought to be in the middle (i.e. Not sure) did not appear in the Table at the Appendix.

These changes do not affect the results or conclusions of the study.
